# Ancient DNA from Giant Panda (*Ailuropoda melanoleuca*) of South-Western China Reveals Genetic Diversity Loss during the Holocene

**DOI:** 10.3390/genes9040198

**Published:** 2018-04-06

**Authors:** Gui-Lian Sheng, Axel Barlow, Alan Cooper, Xin-Dong Hou, Xue-Ping Ji, Nina G. Jablonski, Bo-Jian Zhong, Hong Liu, Lawrence J. Flynn, Jun-Xia Yuan, Li-Rui Wang, Nikolas Basler, Michael V. Westbury, Michael Hofreiter, Xu-Long Lai

**Affiliations:** 1 State Key Laboratory of Biogeology and Environmental Geology, China University of Geosciences, Wuhan 430074, China; glsheng@cug.edu.cn (G.-L.S.); houxd@cug.edu.cn (X.-D.H.); wuhanyjx@126.com (J.-X.Y.); 2 Institute for Biochemistry and Biology, University of Potsdam, Karl-Liebknecht-Strasse 24-25, 14476 Potsdam, Germany; axel.barlow.ab@gmail.com (A.B.); nikolasbasler@gmail.com (N.B.); mvwestbury@gmail.com (M.V.W.); 3 Australian Centre for Ancient DNA, School of Earth & Environmental Sciences, University of Adelaide, Adelaide, SA 5000, Australia; alan.cooper@adelaide.edu.au; 4 Yunnan Cultural Relics and Archaeology Institute, 15-1, Chunmingli, Chunyuanxiaoqu, Kunming 650118, China; jxpchina@foxmail.com; 5 Department of Anthropology, 409 Carpenter Building, The Pennsylvania State University, University Park, PA 16802, USA; ngj2@psu.edu; 6 Jiangsu Key Laboratory for Biodiversity and Biotechnology, College of Life Sciences, Nanjing Normal University, Nanjing 210023, China; bjzhong@njnu.edu.cn; 7 International Joint Research Center for Karstology, Yunnan University, Kunming 650223, China; hongliu@ynu.edu.cn; 8 Department of Human Evolutionary Biology, Harvard University, 11 Divinity Avenue, Cambridge, MA 02138, USA; ljflynn@fas.harvard.edu; 9 Baoshan Museum, Baoshan 678000, China; beyondsdx@sina.com; 10 Natural History Museum of Denmark, University of Copenhagen, Øster Voldgade 5-7, DK-1350 Copenhagen K, Denmark

**Keywords:** ancient DNA, giant panda, evolution, genetic diversity, *Ailuropoda melanoleuca*

## Abstract

The giant panda was widely distributed in China and south-eastern Asia during the middle to late Pleistocene, prior to its habitat becoming rapidly reduced in the Holocene. While conservation reserves have been established and population numbers of the giant panda have recently increased, the interpretation of its genetic diversity remains controversial. Previous analyses, surprisingly, have indicated relatively high levels of genetic diversity raising issues concerning the efficiency and usefulness of reintroducing individuals from captive populations. However, due to a lack of DNA data from fossil specimens, it is unknown whether genetic diversity was even higher prior to the most recent population decline. We amplified complete cyt*b* and 12s rRNA, partial 16s rRNA and *ND1*, and control region sequences from the mitochondrial genomes of two Holocene panda specimens. We estimated genetic diversity and population demography by analyzing the ancient mitochondrial DNA sequences alongside those from modern giant pandas, as well as from other members of the bear family (Ursidae). Phylogenetic analyses show that one of the ancient haplotypes is sister to all sampled modern pandas and the second ancient individual is nested among the modern haplotypes, suggesting that genetic diversity may indeed have been higher earlier during the Holocene. Bayesian skyline plot analysis supports this view and indicates a slight decline in female effective population size starting around 6000 years B.P., followed by a recovery around 2000 years ago. Therefore, while the genetic diversity of the giant panda has been affected by recent habitat contraction, it still harbors substantial genetic diversity. Moreover, while its still low population numbers require continued conservation efforts, there seem to be no immediate threats from the perspective of genetic evolutionary potential.

## 1. Introduction

The giant panda (*Ailuropoda melanoleuca*) is an iconic species for world wildlife conservation. Fossil findings indicate that the ancestors of this species probably originated in the late Miocene in the south-west of China, expanded their habitat range during the early Pleistocene, and began to contract in habitat range during the late Pleistocene [1,2,3,4]. Fossil remains have been excavated from Zhoukoudian in northern China (near Beijing), vast areas of southern China, northern Myanmar, Vietnam, Laos, and Thailand, showing that it was a widely distributed species during the Pleistocene [5,6,7,8]. However, it is currently restricted to only a number of fragmented populations in six different mountain ranges (Qinling, Minshan, Qionglai, Daxiangling, Xiaoxiangling and Liangshan) on the eastern margin of the Tibetan plateau [9]. The dramatic population reduction of this threatened species has attracted concern by conservation biologists and, together with its unique biological attributes such as its coloration or its strictly herbivorous diet (despite being a member of the order Carnivora), it has sparked the interest of evolutionary biologists and population geneticists [10,11,12,13,14,15,16].

Both climate change (Quaternary glacial cycles) and human activities (habitat modification and fragmentation, and hunting) have played important roles in decreasing the genetic diversity and population size of megafauna [17,18,19,20,21,22]. Panda conservation has had a high priority in China since the 1950s and, since 1999, the census in the wild has increased from 1596 to 1864 individuals. Moreover, 71% of wild pandas are now living within the reserve system [9,11]. As a consequence, the International Union for Conservation of Nature (IUCN) has changed the status of giant panda from “endangered” to “vulnerable” [23]. However, the latest national survey (2011–2014) of adult giant panda populations has been improved not only in terms of data collection and analytical methods, but also by increasing the survey area from 49 to 62 counties [24]. Therefore, important questions remain about the benefits of reintroducing more individuals from captive populations versus protecting additional areas of potential habitat. With regard to additional protection zones, it is important to consider habitat quality, the potential for local adaptation of populations, and further climate change [25,26]. In this context, further investigation of past population dynamics and changes in genetic diversity offer the possibility to strengthen and inform management strategies.

Several previous studies have examined the genetic diversity and relationships of extant giant panda populations using both mitochondrial and nuclear data. Early studies using proteins [27], the mitochondrial (mt) DNA control region [28], and DNA fingerprinting [29] have suggested that the genetic variability in giant panda populations is low. In contrast, more recent studies of the mtDNA control region and microsatellite loci suggest that the giant panda has high or at least medium-level genetic variability compared with the other bear species [13,30,31]. This is further supported by genome-wide levels of heterozygosity [14,26].

Although a recent study confirmed the potential for genetic data recovery from ancient giant panda remains [32], ancient DNA sequences that would allow assessment of changes in genetic diversity are yet to be reported. This may reveal additional diversity or entire clades that were lost during the Pleistocene or Holocene [33], as well as contribute to an understanding of changes in population size and genetic diversity over time. In this study, we report mitochondrial DNA sequences comparing the complete cytochrome b (cyt*b*) and 12s rRNA genes, partial 16s rRNA and ND1 genes, and the control region (CR) from two middle Holocene giant panda specimens collected from south-western China. We use these data to investigate the genetic diversity and demographic history of giant panda populations, and discuss the mechanisms behind the giant panda’s range contraction and population size reduction.

## 2. Materials and Methods

### 2.1. Samples

**Figure 1:**
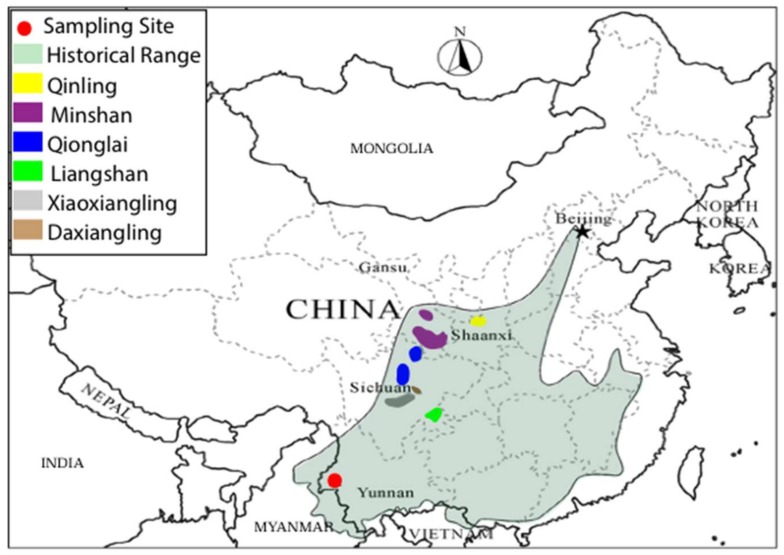
Geographic distribution of the giant panda. The samples in this study are indicated by the red dot. The historical geographic distribution is indicated by light green shading. Different extant populations are shown by different colors, as indicated by the key at the top left of the figure [7,9].

Two samples of Holocene sub-fossil giant pandas (Specimens Nos.: 05001, 97001) were collected from a natural sinkhole, 41–60 m deep from the surface, at Jiangdong Hill, Tengchong County in Yunnan Province, south-western China (Figure 1). The samples were associated with *Elephas maximus*, *Bos gaurus*, *Dicerorhinus sumatraensis*, *Equus yunnanesis*, *Cervus unicolor*, and several other species [34,35,36,37]. They were accelerator mass spectrometry (AMS)-radiocarbon dated at the Quaternary Geology and Archaeological Chronology Laboratory at Peking University at 5025 ± 35 (No. 05001) and 8470 ± 45 (No. 97001) years B.P. respectively [37]. These dates are the latest record of the giant panda before it disappeared from Yunnan Province.

### 2.2. DNA Extraction and Amplification

Ancient DNA extractions and polymerase chain reactions (PCRs) were set up in an ancient DNA facility at China University of Geosciences (Wuhan), in a building physically separated from post-PCR facilities. Ancient DNA was extracted from approximately 250 mg bone powder following a silica method optimized for ancient DNA extraction [38]. Extraction and PCR blanks were performed to monitor for potential contamination. Overlapping primer pairs were newly designed based on the mitochondrial genome of an extant giant panda (GenBank accession number of EF212882.1) [39] using Primer 5.0 (Figure S1 and Table S1). Amplifications were performed using a two-step multiplex approach [40]. The annealing temperatures were set at 50 °C in both steps. PCR products were purified using the QIAquick Gel Extraction Kit (Qiagen, Hilden, Germany) and cloned into the pMD18-T vector (Takara, Tokyo, Japan) following the supplier’s instructions. Plasmids were transformed into competent *E. coli* DH5α. White transformants obtained from LB plates were screened by PCR with the M13 primer pair. For each fragment, a minimum of eight clones, four from each of two independent primary amplifications, were sequenced at Nanjing Genscript Ltd. Company on an ABI 3700 sequencer. When consistent differences were found between the two independent amplifications due to sequence errors, most likely resulting from template damage, a third amplification was performed to determine which sequence was reproducible [41].

Parts of the ancient DNA sequences were replicated at the Australian Centre for Ancient DNA (ACAD) at the University of Adelaide (Supplementary information).

### 2.3. Bioinformatic Analyses

Each fragment obtained was compared to the sequences available in GenBank using NCBI’s online Nucleotide BLAST (https://blast.ncbi.nlm.nih.gov/Blast.cgi, default settings) in order to validate the data. The newly determined DNA sequences were deposited in GenBank (Accession Nos. KF386262-3, KP306766-73).

We also downloaded short read data of 49 modern giant pandas from the European Nucleotide Archive (ENA), accession number SRA053353 [26]. We used Cutadapt v1.4.2 [42] to trim Illumina adapter sequences from all reads and discarded sequences shorter than 30 bp. We used Flash v1.2.10 [43] to merge paired end reads and selected all non-overlapping pairs of trimmed forward and reverse reads, which were then mapped against a reference mitochondrial genome (GenBank accession number of FM177761.1) by using the “aln” and “sampe” algorithms in BWA v0.7.8-r455 with default parameters [44]. We used SAMtools v0.1.19-44428cd [45] to exclude sequences with a map quality score less than 30 (“view”), sort the alignment (“sort”) and collapse sequences with identical 5’ mapping coordinates (“rmdup”). Finally, a consensus sequence was generated for each individual, based on maximum effective read depth [46] using ANGSD v0.916 [47]. Besides assembling the NGS short reads, we retrieved three assembled giant panda mitochondrial genomes from GenBank [39,48,49] which, together with the sequences from our two individuals, provided a total of 54 giant panda mitochondrial sequences. The sequences were aligned with ClustalX v1.81 [50] and the alignments were carefully checked manually. Haplotype identification was carried out by inputting all the variable nucleotide sites among the 54 combined sequences to DnaSP6 [51]. In addition, we aligned the panda sequences with those from 200 additional representatives (4 American black bears, 7 Asian black bears, 2 sloth bears, 120 brown bears, 30 cave bears, 2 spectacled bears, 32 polar bears, 2 sun bears, and 1 American giant short-faced bear) of the family Ursidae [52] and one dog (*Canis lupus familiaris*, GenBank accession number of AB499817.1) as an outgroup for sequence alignment and further phylogenetic analyses (Table S2).

### 2.4. Diversity Comparisons

We subsampled four species or species groups from the aforementioned 254 individuals, which were giant panda, brown bear, Late Pleistocene cave bears, and polar bear, that had a sufficient number of sequences sampled across their respective geographic distributions to compare their genetic diversity. The cave bear sample included sequences assigned to the taxa *ingressus*, *spelaeus* and *eremus*, which may represent different subspecies or species [53]. It should also be noted that brown bear haplotype lineages are paraphyletic with respect to the polar bear clade, as a result of interspecies admixture [54]. We randomly sampled 10 individuals from each group and computed the number of variable positions between each pair, and then computed the average of these pairwise identities. We did this random subsampling and pairwise identity computation 10, 100, and 1000 times. This provided a distribution of the average genetic diversity of these four species or species groups reflecting the variance introduced by the sampling of individuals.

### 2.5. Phylogenetic and Network Analysis

Phylogenetic relationships among the 54 sampled giant panda sequences, as well as their position within the larger Ursid phylogeny, were inferred by maximum likelihood in RAxML-HPC v.8 [55] on the CIPRES portal [56]. *Canis lupus familiaris* served as an outgroup to root the tree. The D-loop sequences were not used for this analysis due to the possibility of saturation and ambiguous alignment of divergent intergeneric sequences. Optimal alignment partitions were selected from all possible combinations of 12s, 16s and the individual codon positions of the protein coding cyt*b* and *ND1* genes under the Bayesian Information Criterion using PartitionFinder v1.1.1 [57] with the greedy search algorithm and linked branch lengths. Only the GTR + G substitution model was considered. Five hundred rapid maximum likelihood bootstrap replicates were carried out using the GTR + CAT substitution model for each partition, which approximates the GTR + G substitution model, with a final maximum likelihood search for the best scoring tree (-f a option). 

To further compare ancient giant panda and previously analyzed extant giant panda haplotypes, we aligned our newly obtained D-loop sequences against the 655 bp D-loop data set from previous studies [13,30]. We used this alignment to reconstruct a median-joining network and thus to investigate the phylogeographical relationship among haplotypes based on maximum-parsimony as implemented in NETWORK v4.6.1.0 [58].

### 2.6. Demographic Inference

Changes in female effective population size over the timespan of the mitochondrial phylogeny were inferred by Bayesian skyline plot analysis [59] in BEAST 1.8.2 [60]. The skyline plot was time-calibrated using the calibrated radiocarbon dates of the two ancient sequences and by applying an informative prior on the per-lineage substitution rate.

The substitution rate for the panda lineage averaged across the 12s, 16s, cyt*b* and *ND1* gene sequences was estimated by aligning sequences from seven living and two extinct representatives of the Ursidae to the sequence of a modern giant panda and computing a time-calibrated phylogeny. Four node calibration priors based on fossils and other evidence were applied, following the approach used in a recent investigation of the Ursidae [61]. The monophyly of each descendent clade was enforced for the analysis. These node calibrations comprised (see [61] and references therein):

1. A uniform prior on the basal divergence of the Ursidae with an upper limit of 12 million years, representing the age of a well-documented fossil representative of the Ailuropodinae, and a lower limit of 20 million years based on a previous molecular dating study.
2. A uniform prior on the divergence of the Tremarctinae and Ursinae clades of 7 to 14 million years, based on the fossil Tremarctine bear *Plionarctos.*
3. A uniform prior on the basal divergence of the Ursinae of 4.3 to 6 million years, based on reported ages of *Ursus minimus.*
4. A uniform prior on the divergence of brown and polar bears of 0.48 to 1.1 million years, based on previous studies. This prior was applied to the common ancestor of a polar bear and a Finnish brown bear, likely representing the initial divergence of these respective species, rather than more recent gene flow events.

Optimal partitions and substitution models were selected as described for the maximum likelihood phylogenetic analysis, considering all substitution models available in BEAUti v.1.8.2 (part of the BEAST v1.8.2 distribution [60]). A lognormal relaxed-clock model was utilized, with an uninformative uniform prior on the mean substitution rate of 0 to 100 percent per million years, both representing implausibly extreme values. The default exponential prior was retained for the ucld.stdev parameter. A speciation birth-death process was used for the tree prior. The Markov Chain Monte Carlo (MCMC) ran for sufficient length to achieve convergence and adequate sampling (ESS > 200) of all parameters as determined using the program Tracer v1.6 [62]. The posterior sample of the lineage-specific substitution rate for the *Ailuropoda* lineage was extracted from the posterior sample of trees using TreeStat v1.8.2 and assessed in Tracer.

Demographic analysis of the 54 sampled giant panda sequences was carried out using a piecewise constant coalescent Bayesian Skyline model with 10 groups. The giant panda D-loop sequences were included in this analysis as they could be aligned unambiguously and substantial saturation is unlikely at the population level. As we failed to recover several sections of the D-loop from the ancient samples, all homologous positions of the D-loop alignment containing missing data were removed. To facilitate the application of the substitution rate for the *Ailuropoda* lineage estimated by the calibrated Ursidae analysis, optimal substitution models were selected for the partitions selected for that analysis with the addition of the D-loop as a separate partition. A single relaxed-clock model was then used for the 12s, 16s, cyt*b* and *ND1* partitions, with a normal prior applied to the per lineage substitution rate approximating the posterior estimate generated by the calibrated Ursidae analysis. A separate, unlinked relaxed-clock model was specified for the D-loop, with the substitution rate estimated within an uninformative, uniform prior between 0 and 2 × 10^−7^ substitutions per site per year. Default exponential priors were used for the ucld.stdev parameters of both relaxed-clock models. The MCMC chain was run as described previously, and the maximum clade credibility tree was extracted and annotated with relevant summary statistics using TreeAnnotator, with node heights scaled to the median of the posterior sample. The Bayesian skyline reconstruction was generated in Tracer.

## 3. Results

### 3.1. Sequence Data Recovery

We amplified 67 overlapping fragments (size range was 53–159 bp excluding primers) from the two Holocene samples. The fragments were used to build the complete cyt*b* (1140 bp) and 12s rRNA (966 bp) genes, partial 16s rRNA (1126 bp for 05001, 1103 bp for 97001), and partial *ND1* (905 bp for 05001, 923 bp for 97001) genes, and D-loop sequences (1052 bp for both specimens) (Figure S1 and Table S2). Identical sequences were obtained when experiments were independently replicated at the State Key Laboratory for Biogeology and Environmental Geology at China University of Geosciences (Wuhan) and the Australian Centre for Ancient DNA at the University of Adelaide, Australia.

### 3.2. Diversity Comparisons

**Figure 2:**
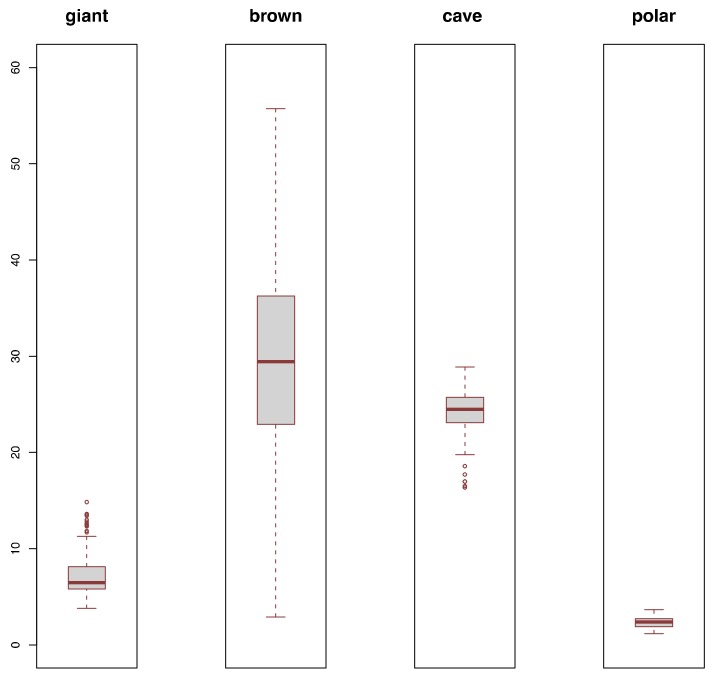
Genetic diversity distribution of four ursid species or species groups. Boxplots summarize the mean pairwise number of variable positions (*Y* axis) from 100 random subsamples of 10 individuals.

Average pairwise substitutions for brown bear haplotypes were highly variable and included by far the highest diversity values observed for any group. Late Pleistocene European cave bears, despite covering several tens of thousands of years, showed less variance in mean substitutions across replicates, with values falling within those observed for brown bears. Polar bear haplotypes were highly similar with little apparent variation introduced by sampling. Diversity estimates for giant panda haplotypes were generally lower than observed for brown bears, always lower than observed for Late Pleistocene European cave bears, but always higher than observed for polar bears (Figure 2 and Figure S3).

### 3.3. Phylogenetic and Network Analysis

**Figure 3:**
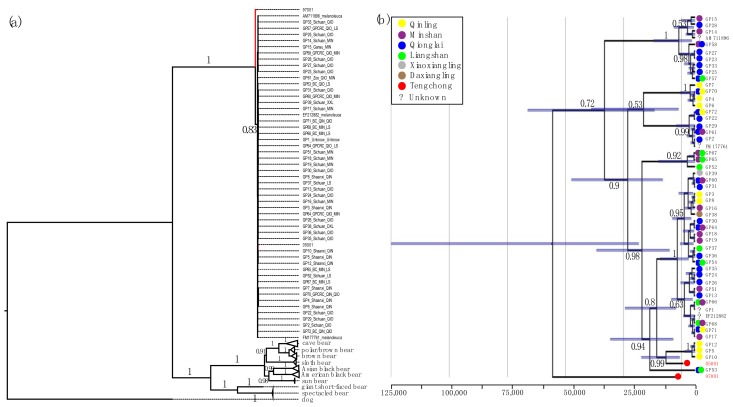
Phylogenetic relationships of the extant bear family based on mitochondrial DNA sequences. (**a**) Maximum likelihood phylogenetic tree inferred from four aligned bear mitochondrial genes. The giant panda clade is expanded while other clades are collapsed. Red branches in the giant panda clade indicate the two ancient panda individuals. Bootstrap proportions for major clades are indicated (**b**) Time-calibrated phylogeny of giant panda clade, based on the same four genes plus the mitochondrial control region, using the same color codes as in Figure 1. Double color-filled circles next to individual names indicate haplotypes recovered from different populations. Question marks indicate individuals for which geographic information is not available. The lower scale shows years before present. Branch labels indicate posterior clade probabilities, except for some terminal tips where labels have been removed for simplicity. Nodes are centered on median estimated coalescence times from the posterior sample, with bars indicating the 95% credibility interval.

A total number of 71 variable nucleotide positions (Figure S2), corresponding to 20 haplotypes (Table S3), were identified in the 54 giant panda data set that included 12s, 16s, cyt*b*, and *ND1* sequences. The optimal data partitions selected by PartitionFinder are shown in Supplementary Tables S4 and S5. Maximum likelihood analysis placed the *Ailuropoda* mitochondrial lineage as sister to all other sampled ursid lineages with strong bootstrap support (100% of replicates, Figure 3). The ancient individual 97001 is sister to the clade containing all modern panda haplotypes and the second ancient individual, sample 05001. Although this relationship received strong bootstrap support (83% of replicates), relationships within the latter clade were largely unresolved by this analysis (occurring in <70% of bootstrap replicates for many clades).

**Figure 4:**
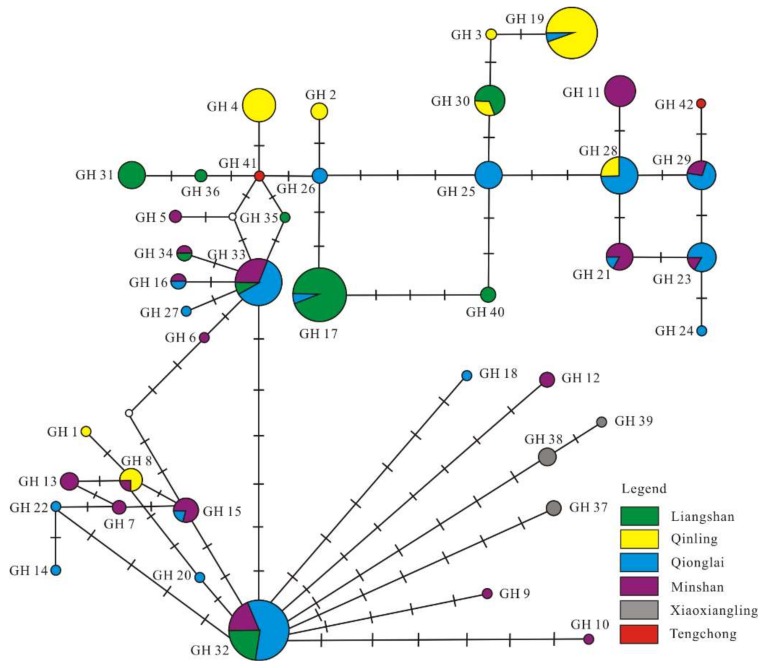
Median-joining network of the 655 bp mitochondrial DNA control region haplotypes based on the combined data of the Holocene sub-fossil giant pandas and extant populations presented by Hu et al. [30]. Different color codes represent different populations in terms of mountain ranges. Circles are sized relative to haplotype frequency. Tick marks along lines indicate the number of nucleotide substitutions between haplotypes. The definition of the haplotype names follows Hu et al.

A network analysis of the two Holocene giant panda D-loop sequences with 40 homologous published extant panda sequences (from 169 individuals, 655 bp) retrieved a network profile of 42 haplotypes (GH 1–42, Figure 4, Table S2), with the two Holocene samples forming two new different haplotypes GH41 (05001, GenBank accession #KF386262) and GH42 (97001, GenBank accession #KF386263). A total number of 18 variable sites in the Holocene giant pandas have been identified among the 655 bp data set (Table S6). Two out of 18 variable sites in GH42 were unique, while 18 variable sites in GH41 and 16 out of 18 variable sites in GH42 were shared with those of modern giant pandas. The rest of the network shows the same configuration as previous studies, with haplotypes GH32 and GH33 as the central sequences in the two-star like network profiles [13,30]. High haplotype (Hd = 0.9572 ± 0.0171) but relatively low nucleotide diversity (π = 0.0054 ± 0.0003) mark the mitochondrial DNA diversity of the giant panda.

### 3.4. Demographic Analysis

Partitions and substitution models used in the BEAST analyses are shown in Supplementary Tables S4 and S5. The calibrated Ursidae analysis returned a unimodal skewed approximately normal posterior distribution for the lineage specific *Ailuropoda* substitution rate averaged across the 12s, 16s, cyt*b* and *ND1* genes, with a median value of 3.3954 × 10^−8^ substitutions/site/year. Based on this result we applied a normal prior on the per lineage substitution with a mean value of 3.3954 × 10^−8^ substitutions/site/year and standard deviation of 7.705 × 10^−9^ substitutions/site/year to the corresponding partitions in order to calibrate the Bayesian skyline analysis.

Congruent with the maximum likelihood analysis, the maximum clade credibility tree generated by Bayesian skyline analysis returned the sequence of the ancient sample 97001 as sister to all other sampled panda haplotypes with a posterior clade probability 0.72 (Figure 3). Many relationships within the giant panda clade were better resolved (posterior clade probabilities >0.95) than in the maximum likelihood analysis. This included the position of the ancient sample 05001, placed within the diversity of modern lineages as sister to a clade comprised of three haplotypes sampled in Shaanxi Province (posterior clade probability 0.99).

**Figure 5:**
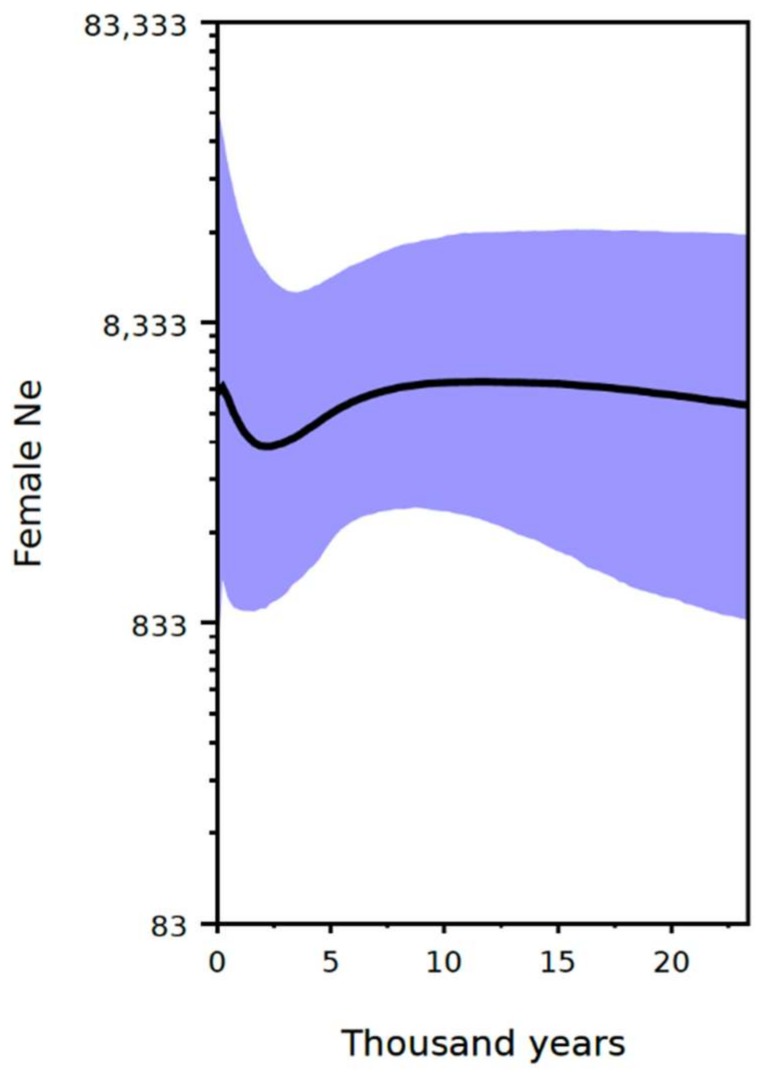
Bayesian skyline demographic reconstruction of female effective population size (Ne, *y* axis, note log scale) through time (*x* axis), assuming a generation time of 12 years [26]. Black line indicates median female Ne, and the shaded blue area indicates the 95% credibility interval.

The applied tip and substitution-rate calibrations returned a median coalescence time for all sampled haplotypes of ~62,000 years (95% credibility interval ~23,000 to ~131,000 years). The median estimated coalescence time for sampled modern haplotypes is ~39,000 years (95% confidence interval 21,000 to 73,000 years). The Bayesian skyline reconstruction showed a decline in female effective population size around 6000 years ago, followed by population recovery during the last 2000 years (Figure 5).

## 4. Discussion

### 4.1. Genetic Diversity and Demographic History of Giant Pandas

Large-scale environmental alteration due to influences such as climate change or human activities has caused many formerly widespread species to survive in small fractions of their original range. These range contractions have often been accompanied by a loss of intraspecific genetic diversity due to the reduction of effective population size and the extinction of regional genetic lineages [63]. Giant pandas are now restricted to fragmented habitats in Sichuan, Gansu, and Shaanxi Provinces [8]. However, previous studies have suggested that the two largest populations of giant panda, the Minshan and Qionglai populations, as well as a smaller Liangshan population, nevertheless have retained relatively high levels of genetic diversity despite a trend of recent population decline [13,30]. These previous studies also showed that the haplotypes of the Minshan, Qionglai, and Liangshan populations are distributed broadly while the Xiangling and Qinling populations are more localized.

The mid-Holocene giant panda analysed in this study represent two new haplotypes in the network profile based on a 655 bp D-loop dataset (Figure 4). While one haplotype is, in phylogenetic reconstructions, nested within the genetic diversity of modern panda haplotypes, the other haplotype is sister to all other sequenced pandas (Figure 3). The probability that the deepest divergence in a panmictic population has been sampled can be estimated as (n − 1)/(n + 1), where n represents the number of individuals [64]. For 52 modern individuals, this probability is 0.96, meaning that, under the assumption that the fossil giant panda belongs to the modern population, it is highly unlikely that a sequence such as the one from sample No. 97001 in our analysis can be retrieved. Moreover, the sequence from this sample is quite divergent from all other sequences included in this study. When excluding individual No. 97001, we find the number of polymorphic positions in the combined data set (includes 12s, 16s, cyt*b*, and *ND1* fragments (4054 bp)) to be 36. However, this increases to 71 when individual No. 97001 is also included. The giant panda probably disappeared from Yunnan Province approximately 5000 years ago as the dates of our samples represent the latest record of its appearance in this area. The extinction of this population appears to have resulted in a loss of distinct mitochondrial lineages as seen in other species [21,33]. A loss or turnover of mitochondrial haplotypes over time has been observed in several carnivore species. For example, in North America, the northern brown bear population, representing a separate haplogroup, disappeared before 35 ka B.P. and was replaced after 21 ka B.P. by one of the modern haplogroups [65]. A more immediate replacement was observed for cave bears in southern Germany (in the Ach valley), where the original haplogroup overlapped temporally with the incoming haplogroup that eventually replaced the initial one around 30,000 years ago [66,67]. In contrast to the Holocene loss of the divergent haplotype in the giant panda, both of these replacements took place during the Pleistocene. This is also true for the loss of haplogroup 2 in North American wolves at the end of the Pleistocene [68]. However, further analyses showed that in Eurasia, haplogroup 1 continued to displace haplogroup 2 during the Holocene [69], but without a concomitant loss of geographical distribution. The example most similar to our results from the giant panda—a loss of a divergent haplotype or group co-occuring with the disappearance of a local population—stems from European brown bears. This was found in brown bears from southern France, where three distinct and related haplotypes disappeared with the loss of the southern France brown bear population between 1570 to 6525 years B.P. [70,71]. However, in contrast to our findings from the giant panda, these haplotypes did not represent the deepest diverging lineages within the mitochondrial DNA tree. Thus, even though the giant panda is a successful species highly adapted to its specialized diet via morphological, ecological, and genetic adaptations [32], one should keep in mind that the local extinction in geographic locations such as Yunnan Province may not only have led to a reduction of overall population size but also to a vanishing of distinct and divergent mitochondrial clades.

However, despite the deep divergence of one of the fossil individuals and although neither of the two ancient haplotypes have been found in modern giant panda populations, the high haplotype diversity observed suggests that the range losses for giant pandas, such as the local extinction at the sampling site in this study, and recent reduction in wild population size have not resulted in genetic impoverishment during the past 8000 years. Historical distribution data show that as recent as the sixteenth to nineteenth centuries, giant pandas were still widely distributed in south-western China, including Hunan, Hubei, Sichuan, Shaanxi, and Gansu Provinces [6,72]. This historical habitat status indicates that the range contractions and decrease in population size may have been too recent to show marked genetic impacts. Therefore, the giant panda appears to be an example of a species showing temporal lag between a pronounced reduction in population size and the genetic impacts of that reduction in terms of mitochondrial DNA diversity.

### 4.2. Impacts of Human Activities and Climate Change

Although modern giant panda populations display moderate genetic diversity, a previous study suggested that the giant panda population experienced two recent bottlenecks at ~0.2 million years and ~20,000 years ago, respectively [24]. However, the more recent panda demographic history younger than 20,000 years could not be evaluated by the pairwise sequentially Markovian coalescent (PSMC) approach applied in that study. Using our data set consisting of both modern and ancient panda mitochondrial DNA sequences, we were able to reconstruct the Holocene demographic history of giant pandas. Our results suggest a slight decrease around 6000 years ago and a recovery to the original population size about 2000 years ago, although it has to be noted that the confidence intervals of this estimate are fairly large (Figure 5). Therefore, we found no evidence for a marked reduction of female effective population size using our data.

Both global climate change and human activities have been cited as key factors shaping giant panda population demography [73,74]. The periods of the two identified bottlenecks coincide with peaks in accumulation of loess, indicating cold and dry periods in China [75]. This suggests that periods of climate change might have resulted in extensive loss of giant panda habitats, most likely due to a reduction of bamboo availability, the almost exclusive food source of giant panda. This is in line with recent suggestions of the role of climate change in megafaunal genetic transitions [21,76,77]. Interestingly, we found no evidence for a population decline during the last glacial maximum, but this could be due to the more recent population bottleneck erasing the signal of earlier demographic events from the mitochondrial locus. One might expect that the warming climate since the beginning of the Holocene may have resulted in an increasing panda population size after the last glacial maximum. However, encroachment of human populations into giant panda habitat may have prevented a population recovery.

The reduction in female effective population size from 6000 to 2000 years B.P. in our skyline plot does coincide with the appearance of a cold period in south-western China (Figure 5). It has been implied that summer monsoon precipitation changed asynchronously over East Asia during the Holocene, decreasing earlier in southern China and later in northern China [78]. Boreal pollen studies have indicated that there was a continuing and extensive decline in forest habitats in northern China around 4000 years B.P. [79]. The δ^13^C time series of the *C. mulieensis* remains and mixed plant cellulose of the Hongyuan peat in south-western China reflects that the Indian Ocean summer monsoon precipitation declined during 4200–4000 years B.P. [80]. The δ^18^O record of the Dunde ice core from Qilian Mountain in western China suggested that the lowest temperature was detected in that area in the same period. This period was also the time when Dayu regulated floodwater in southern China according to historical records, suggesting high precipitation during this cold period [81]. It is difficult to invoke human influences in this time period. However, archeological studies have shown that revolutionary improvements in farming technology occurred at the beginning of the Spring and Autumn Period in China (770–486 BC) [82]. The northern Qinling region was not extensively settled until the last 2.5 ky [83]. At Liangshan Mountain, it has been reported that agricultural activity expanded alongside a growing resident human population approximately 1000 years ago. Because agriculture came to Yunnan Province relatively late due to the steep topography and high altitude, it appears that anthropogenic disturbances were not critical in south-western China at that time.

While it seems that the giant panda individuals in this study fell into the sinkhole while the area was still richly forested, the fact that their bones had been washed into their positions, and that the soil deposits in the floor in the sinkhole revealed flooding events and soil erosion, could suggest local deforestation by humans [38]. Later on, there is increasing evidence of human activities, especially during the last 600 years. An early to middle Ming Dynasty bowl was found in the cave fill below the mouth of the sinkhole [36]. Unequivocally, in the early period of the Ming Dynasty, soldiers had been dispatched as residential agricultural armies to inhabit and undertake farming in frontier areas such as Yunnan that had previously been governed by other polities. Moreover, an acceleration of habitat loss and degradation occurred during the middle Qing Dynasty (1723–1820 AD) when highly productive crops such as maize and potato were widely planted [84]. As such, the rapid range contraction and fragmentation of giant panda populations caused by human activities would appear to be a relatively recent phenomenon. This interpretation is supported by our genetic data, which fail to reveal any substantial decreases in effective population size.

The mitochondrial sequence data set used here has already provided clues with regard to population size and genetic diversity through time. Larger sequence datasets, such as the whole genome sequencing of Holocene giant panda individuals, will likely allow further identification of the extent of genetic diversity loss and hopefully reveal key factors involved in the contraction of giant panda population and range sizes.

## Supplementary Materials

The following are available online at http://www.mdpi.com/2073-4425/9/4/198/s1, Figure S1: Schematic view of the complete or partial mitochondrial genes for the Holocene giant panda specimens using overlapping PCR fragments. Figure S2: Variable nucleotide positions in the 4054 bp data set of 12s, 16s, cyt*b*, and *ND1* fragments from 54 giant panda individuals. Figure S3: Genetic diversity distribution of the four subsampled species when random subsampling and pairwise identity computation have been performed 10, 100, and 1000 times, respectively. Table S1: PCR primers for giant panda mitochondrial genes. Table S2: Sequence information used in this study. Table S3: Haplotypes identification of 54 giant panda individuals used for phylogenetic analyses in Figure 3. Table S4: Optimal data partitions and substitution models selected by partitionfinder for the BEAST analyses. Table S5: Optimal data partitions selected by partitionfinder for maximum likelihood phylogenetic analysis. Table S6: Variable sites in the aligned 655 bp D-loop data set.
