# Supplementary material for: Ancient DNA from Giant Panda (Ailuropoda melanoleuca) of South-Western China Reveals Genetic Diversity Loss during the Holocene
Source: Genes (Basel). 2018 Apr 6;9(4):198. doi: 10.3390/genes9040198 (PMC5924540; doi:10.3390/genes9040198)
Supplement: Supplementary file 1 [file genes-09-00198-s001.pdf]

1. DNA Sequencing Replication

Independent replication of the ancient DNA sequences was done at the Australian Centre for Ancient DNA (ACAD) at the University of Adelaide. Ancient DNA was extracted from two Holocene samples using a Qiagen Blood & Tissue Kit (Valencia, California) and a modified protocol with added EDTA and Proteinase K [85]. PCR amplifications were carried out in 25 μL volumes, using 1× PCR buffer, 2.5 mM of $MgSO_4$, 1 mg/mL of rabbit serum albumin (RSA), 0.2 μM of each primer, 0.25 mM of dNTPs, 1 U of Platinum HiFiTaq (Invitrogen) and 2 μL of ancient DNA extract. The PCR was started with an initial denaturation at 94 °C for 2 min, followed by 50 cycles of denaturation at 94 °C for 15 s, annealing at 52 °C for 30 s, and extension at 68 °C for 30 s. The last step was a 10 min final extension period at 68 °C. PCR products were visualized under UV light on a 3.5% agarose gel stained with ethidium bromide. Successful amplifications were purified using Ampure (Agencourt) according to the manufacturer's instructions. Both DNA strands were sequenced directly using Big Dye chemistry and an ABI 3130XL Genetic Analyzer (Applied Biosystems). For both specimens, a total number of 488 bp of the cyt*b* gene, 374 bp of the 12s rRNA gene, and 469/ 470 bp of the D-loop were independently replicated at the Australian Centre for Ancient DNA (indicated by * in Table S1).

2. Authenticity of DNA Sequences

The following points support the authenticity of the newly obtained Holocene giant panda sequences: (a) DNA extractions and pre-PCR procedures were carried out in an isolated ancient DNA laboratory, PCR amplifications and post-PCR analyses were carried out in another physically separated laboratory; (b) both extraction and PCR blanks were conducted throughout the study and the results were consistently negative; thus there was no contamination from the reagents or environment; (c) fragments were amplified in several overlapping PCRs from multiple extractions, amplifications and multiple colonies were sequenced for each product. No mosaic haplotypes occurred in the two sequences, which suggests there was no cross contamination; (d) no modern giant panda samples have been studied in either of the both ancient DNA laboratories; (e) partial sequences have been successfully repeated at Australian Centre for Ancient DNA in Adelaide, Australia; (f) the two ancient haplotypes have never before been observed.

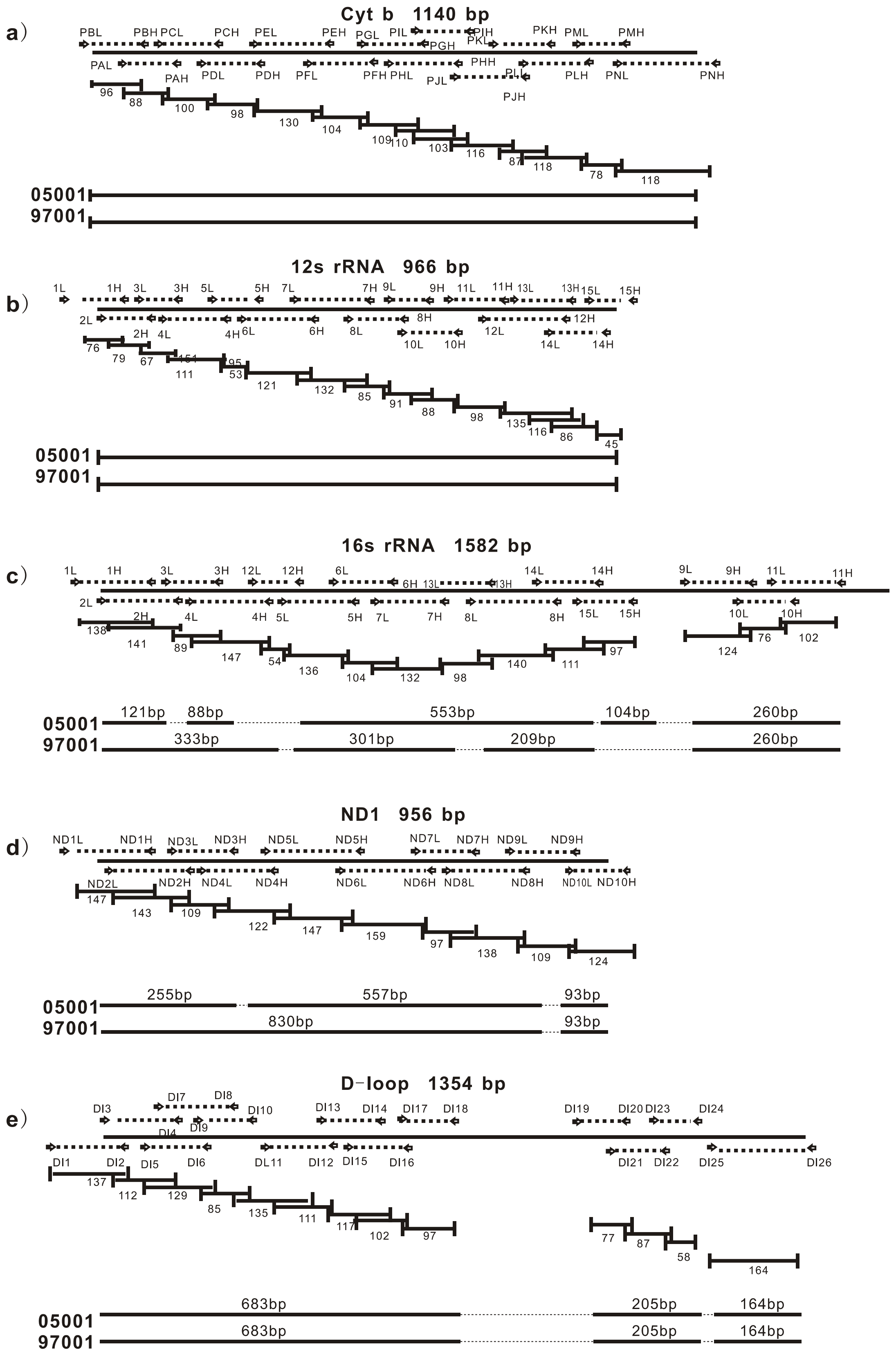

Figure S1. Schematic view of the complete or partial mitochondrial genes for the Holocene giant panda specimens using overlapping PCR fragments. (**a**) cyt*b*; (**b**) 12s rRNA; (**c**) 16s rRNA; (**d**) *ND1*; (**e**) D-loop. Numbers below fragments show length of the amplification products without primers.

```
                  10        20        30        40        50        60        70
          ....|....|....|....|....|....|....|....|....|....|....|....|....|....|.
FM177761  ACNTCTGCCGCCCGCTTTGCCTTCTTCAACTTCCTACGGCCAGCATAAGACCCTCCTAAGCACACTGAACT
05001     .T.........T......A...C...T.....T...T..........G............T...T......
97001     CT.CTC.TTA..TATC..A....TC...G.C...C...A.TGA......GTT.C.T...A.GT.T.....C
GP1       ...................T........................G..G....T.......T....N.N...
GP2       ...................................................................N...
GP3       ...................T........................G..G............T......N...
GP4       ................C..........G.T.....G..................T.C..........N...
GP5       ....................................T......A...G............T......N...
GP6       ................C..........G.T.....G..................T.C..........N...
GP7       ................C..........G.T.....G...T..............T.C........N.N...
GP8       ...................T........................G..G............T......N...
GP10      ....................................T......A...G............T.....NN...
GP12      ....................................T......A...G.........N..T......N...
GP13      ...................T........................G..G............T....N.N...
GP14      .................C.....T.....................C..A................CNN...
GP15      ......A..........C.....T.....................C..A................C.N...
GP16      ...................T........................G..G..........T.T......N...
GP17      ...................T........................G..G....T....N..T....N.N...
GP18      ...................T........................G..C............T......N...
GP19      ...................T........................G..C............T....N.N...
GP22      ...................................................................N...
GP23      .................C.....T.........T...........C..A................C.N...
GP24      ...................T........................G..G.........N..T....N.N...
GP25      ..A..............C.....T.....................C..A................C.N...
GP26      ...................T........................G..G............T..N...N...
GP27      .................C.....T.........T...........C..A................C.N...
GP28      .................C.....T.....................C..A................C.N...
GP29      ...................................................................N...
GP30      ...................T........................G..G............T......N...
GP31      ...................T.................A......G..G............T......N...
GP33      ..A.......A......C.....T.....................C..A................CN....
GP35      ...................T...........C............G..G.........N..T..........
GP36      ...................T.....N..................G..G............T..........
GP37      ...................T........................G..G............T....N.N...
GP38      ...................T........................G..G............T......N...
GP39      ...................T.................A......G..G............T.....NN...
GP51      ...................T........................G..G.........N..T......N...
GP52      ...............................................G.........N........NN...
GP53      ..............................................GG.........N..T......N...
GP54      ...................T........................G..G.........N..T......N...
GP57      ..A..............C.....T.....................C..A................C..N..
GP58      .................C...A.T.....................C..A................C.N...
GP60      ...................T.................A......G..G............T......N...
GP61      .................................................................N.N...
GP64      ...................T........................G..G............T......N...
GP65      ...............................................G.................NNN...
GP66      ...................T........................G..G....T.......T....N.N...
GP67      ...............................................G.........N.........N...
GP68      ...................T........................G..G....T....N..T....N.N.N.
GP70      ................C..........G.T.....G..................T.C.........NN...
GP71      ...................T........................G..G....T....N..T....NNN...
GP72      ..................................................................NN...
AM711896  .................C..T..T.....................C..A................C.....
EF212882  ...................T........................G..G....T....G..T..........
```

Figure S2. Variable nucleotide positions in the 4054 bp data set of 12s, 16s, cyt*b*, and *ND1* fragments from 54 giant panda individuals.

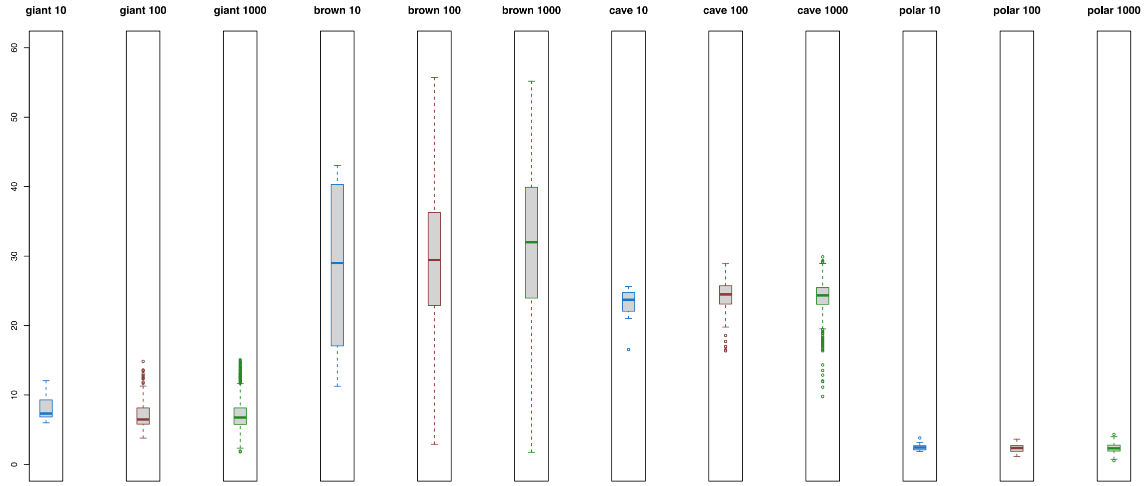

Figure S3. Genetic diversity distribution of the four subsampled species when random subsampling and pairwise identity computation have been performed 10, 100, and 1000 times, respectively.

Table S1. PCR primers for giant panda mitochondrial genes.

Gene Name	Primer Name	Primer Sequence (5′-3′)	Amplicon Size	Multiplex Set
Cyt*b*	PAL<br>PAH	ATTGACCTTCCAACACCATC<br>GTGTGCGACTGATGAAAAGGCT	150 bp	1
	PBL<br>PBH	TCAACTATAAGAACCCCA<br>CCTAACAGGGACCCAAAG	132 bp	2
	PCL<br>PCH	TCCCTGTTAGGAGTGTGTCT<br>ATCGGATAAATCACCCATA	139 bp	1
	PDL *<br>PDH *	GCTACAGCCTTTTCATCAGTC<br>GATCCATAGTATAAGCCTCGC	140 bp	
	PEL *<br>PEH *	TATCTGCCTATTTATACACG<br>AATGACGGTTGCTCCT	166 bp	2
	PFL *<br>PFH *	GGGTATGTACTACCTTGAG<br>CGGTTAGTGTTGCTTTATCT	143 bp	1
	PGL *<br>PGH *	GGGGTTTCTCCGTAGATAAAGC<br>ATCTGGGTCAGATGGAATTCCG	152 bp	2
	PHL *<br>PHH *	CTCAGCACTAGCAATAGTCC<br>AGGCGAGGACAAGAAATAGG	149 bp	1
	PIL<br>PIH	ATAACCCCTCCGGAATTCCATC<br>TCAGGGTCTCCTAACAGGTCT	145 bp	2
	PJL<br>PJH	AACCCTGGCTTTATTCTCAC<br>GCACCCCTCCTAGTTTATTA	155 bp	1
	PKL<br>PKH	GCCTACGCTATCCTGCGATCT<br>AGGTCGGAATATCATGCTTCGT	129 bp	2
	PLL<br>PLH	GGGGTGCTAGCTCTAATCTTC<br>CTGGCTGTCCTCCAATTCATGT	160 bp	1
	PML<br>PMH	TCCGACCTCTAAGTCAATGC<br>GAGGCCAATTGCCCAATA	139 bp	2
	PNL<br>PNH	GAATTGGAGGACAGCCAGTA<br>TGTTGCTCCTTCCTTGAGTC	219 bp	1
12s rRNA	12s-1L<br>12s-1H	AGGTACTGAAAATGCCTAGATGG<br>GCATTTTCACTGGAGCGTGGAGA	120 bp	2

	12s-2L *<br>12s-2H *	GGTTTGGTCCTAGCCTTCCT<br>GTGCTTGATACCCGCTCCTT	118 bp	1
	12s-3L *<br>12s-3H *	ATGTAAGTCTCCACGCTCCA<br>AGCAAGGCGTTGTGAGC	103 bp	2
	12s-4L *<br>12s-4H *	AGGAGCGGGTATCAAGCACA<br>GTGGCTGGCACGAGATTTAC	151 bp	1
	12s-5L *<br>12s-5H *	CGACTAAGCTATGTTAATACTAGGG<br>CGCTTTACGCCGTAGGC	95 bp	1
	12s-6L *<br>12s-6H *	CCGCGGTCATACGATTAACTCGAGT<br>GGCCCTAGCTATCGTGTAATCAGAA	171 bp	2
	12s-7L<br>12s-7H	GGCGTAAAGCGTGTAAAAGA<br>TTAGAGCTAGGCATAGTGGG	172 bp	1
	12s-8L<br>12s-8H	TCTGATTACACGATAGCTAGGGCCC<br>CCAAGTCCTTTGAGTTTTAAGCTGT	135 bp	2
	12s-9L<br>12s-9H	ACTAAACAAAACTATTCGCCAGAG<br>TTAGCAAGGGATGGTGAGGT	135 bp	1
	12s-10L<br>12s-10H	AACAGCTTAAAACTCAAAGGACTTG<br>AAGGGTTTGCTGAAGATGGCGGT	136 bp	2
	12s-11L<br>12s-11H	GATAGACCTCACCATCCCTTGC<br>CCATTTCTTCCCAGCCCATA	139 bp	1
	12s-12L<br>12s-12H	CATAAAAAAGTTAGGTCAAGGTGTA<br>GGGTGACGGGCGGTGTGTGCGTGCT	185 bp	2
	12s-13L<br>12s-13H	TGGGCTGGGAAGAAATGGGCTAC<br>AGGAGGGTGACGGGCGGTGT	139 bp	1
	12s-14L<br>12s-14H	TTGAACAGGGCAATGAAGCACG<br>CCAGTATGCTTACCTTGTTA	127 bp	2
	12s-15L<br>12s15H	GCAAAGCATAAGAGGAGACAAGTCG<br>AAGCCAGACGCTTTGTTTAAGCTAC	95 bp	1
16s<br>rRNA	16s-1L<br>16s-1H	GGCTTACATCCAGAAGATTTCATTA<br>CTTTCATCATTCCCTTGCGGTA	185 bp	2
	16s-2L<br>16s-2H	CAAAGCTAGCCCAAGCAACAATGAC<br>ATACAAAAGGTAGAAGGGGCAATC	189 bp	1
	16s-3L<br>16s-3H	CTATAGAGAGAGTACCGCAAGGGAA<br>TCGGGGGACTTAGCTTAAGTTCT	136 bp	2
	16s-4L<br>16s-4H	ATTGCCCCTTCTACCTTT<br>CACCAGGCTCGTTAGGC	181 bp	1
	16s-12L<br>16s-12H	AGTGAGAAGATCCATAGGTAGAGGT<br>AGTTTTGTAGGCAGGTTTAAAGTCG	103 bp	2
	16s-5L<br>16s-5H	CGAGCCTGGTGATAGCTGGTTGCC<br>GCCTCTAGGCCTACTATGGCTTATA	182 bp	1
	16s-6L<br>16s-6H	TACAGCTTTTTAGAGTTAAGGATAC<br>CATTAGGAGTTGATTAAATATTCTT	153 bp	2
	16s-7L<br>16s-7H	GGCCTAGAGGCAGCCATCAATTAAG<br>TGCTGTTATAAGCTTATGCATGGGA	179 bp	1
	16s-13L<br>16s-13H	CTCCTAATGTATTACTGGGTCAATC<br>CATTAGGTTATTTCTATGTTGTTGT	146 bp	2
	16s-8L<br>16s-8H	CAGCAACGGATAACCACTGATAG<br>AATACTGGAAATGCTGGAGGTGATG	187 bp	1
	16s-14L<br>16s-14H	GGCAAATACAAACCCCGCCTGT<br>TCATACAAGTCCTTATTTAGAGAAC	157 bp	2
	16s-15L	TATTCTGACCGTGCAAAGGTAGCAT	146 bp	1

	16s-15H	TCGTCTTATTGTCTTATCCCCGCCT		
ND1	ND1L<br>ND1H	GTGTCCAGAGGTTCAATTCC<br>GCATCTGCGATTGGTTGTAG	187 bp	2
	ND2L<br>ND2H	CAGCTTCGAAAAGGCCCAAA<br>CGGTATTGGTAAGGGGACTCA	183 bp	1
	ND3L<br>ND3H	GCCCCTATTCTAGCCTTAACAC<br>TGAGGCTCATCCAGATCAGA	150 bp	2
	ND4L<br>ND4H	ACCATGTGAGTCCCCTTACCAA<br>ATGAGATCGTTTGAGCTACGGC	175 bp	1
	ND5L<br>ND5H	GATGAGCCTCAAACTCAAAA<br>CTAGTGGTCATGCAGGGAAG	187 bp	2
	ND6L<br>ND6H	CGCTATCCACGCTAATCA<br>TAGGAAGAACAGGGCAAA	194 bp	1
	ND7L<br>ND7H	CTCTGGGTTTAATGTCGA<br>GGTATGTAGGGGCTGTGA	132 bp	2
	ND8L<br>ND8H	TAGCAGAATATGCCAACATC<br>TCGTATCGGAATCGAGGA	175 bp	1
	ND9L<br>ND9H	TTTCTATGAATCCGAGCA<br>GTTTGAGGTGGAATGCTTGC	146 bp	2
	ND10L<br>ND10H	CGACCAACTAATACACCTA<br>CCCCTATGGCTTACTCTA	161 bp	1
D-loop	DL-1<br>DL-2	GGAAGGAGCAACAACCCCACTACCA<br>GAAAACATACTATGATGGCACAGAG	138 bp	2
	DL-3<br>DL-4	TCCACCTCTCATTTTATTCACTTCA<br>GGGGCACGCCATTAATGCACGATA	160 bp	1
	DL-5<br>DL-6	GTATGTTTTCATACATCCTCCCT<br>CGCTTGAAATAAGTTCTTGTAATG	176 bp	2
	DL-7 *<br>DL-8 *	TGTGCTTGGCTTTACATGAGG<br>CTCGCAAGGATTGCTGGTTT	126 bp	1
	DL-9 *<br>DL-10 *	TATTTCAAGCGATAGTCTATGAGC<br>TGGCCCTGAGGTAAGAAC	177 bp	2
	DL-11 *<br>DL-12 *	TGCGAGTACGTGTACCTCTTC<br>ATTAGTCCATCGAGATGTCCC	153 bp	1
	DL-13 *<br>DL-14 *	CAACTCAATCCTACTAACCCTTCA<br>ACTGCGACGAGACCTTTACG	161 bp	2
	DL-15 *<br>DL-16 *	TTTTAGGGGGGGAACTTGCTATGAC<br>TAGTGTTATGTCCTGTGACCATT	150 bp	1
	DL-17 *<br>DL-18 *	GTCTCGTCGCAGTCAAATCA<br>CGTACACGTAACGCCAGTCC	137 bp	2
	DL-19<br>DL-20	TAAGTTAGCTTAGACAAACCCCCC<br>GGTGTAAGTGACTTCATGTTTGCGC	126 bp	1
	DL-21<br>DL-22	TGGATGTCCTGCCAAACC<br>AGTCCCATGTCCGTAGTTGTT	126 bp	2
	DL-23<br>DL-24	ATAATTAAGCTAACCCCCCAGCCAA<br>CAATGCTGGGGAGACACAGTAAAA	107 bp	1
	DL-25<br>DL-26	TGTGTCTCCCCAGCATTGATTTTTT<br>GGTAGCGTGACCCATCTAGGCATTT	150 bp	2

* Primer pairs have been replicated at ACAD.

Table S2. Sequence information used in this study.

No. in This Study	Species	GenBank Accession No.	Length	Reference
97001	*Ailuropoda melanoleuca*	KP306773 (cyt*b*)	1140 bp	This study
		KP306768 (12s rRNA)	966 bp	This study
		KP306766 (16s rRNA)	1103 bp	This study
		KP306770 (ND1)	923 bp	This study
		KF386262 (D-loop)	1052 bp	This study
05001	*Ailuropoda melanoleuca*	KP306772 (cyt*b*)	1140 bp	This study
		KP306769 (12s rRNA)	966 bp	This study
		KP306767 (16s rRNA)	1126 bp	This study
		KP306771 (ND1)	905 bp	This study
		KF386263 (D-loop)	1052 bp	This study
AM711896	*Ailuropoda melanoleuca*	AM711896	16,846 bp	[47]
EF212882	*Ailuropoda melanoleuca*	EF212882	16,805 bp	[38]
FM177761	*Ailuropoda melanoleuca*	FM177761	16,796 bp	[48]
NC009492	*Ailuropoda melanoleuca*	NC_009492	16,805 bp	[38]
EF100819-57	*Ailuropoda melanoleuca*	EF100819	655 bp	[13]
SRA053353	*Ailuropoda melanoleuca*	SRA053353	2.25 Gb	[26]
KX641289-337	*Ursus spelaeus/Ursus arctos*	KX641289-337	~16,300 bp	[50]
AP012559-97	*Ursus arctos/Ursus maritimus*	AP012559-97	~16,900 bp	[86]
NC_003426-28	*Ursus americanus Ursus arctos Ursus/maritimus*	NC_003426	~16,840 bp	[48]
FM177762-65	*Ursus thibetanus/Melursus ursinus/Tremarctos ornatus*	FM177762-65	~16,800 bp	[48]

Table S3. Haplotypes identification of 54 giant panda individuals used for phylogenetic analyses in Figure 3.

Haplotype Nos.	Sample Nos. In Figure 3(b)
Hap_1	05001
Hap_2	97001
Hap_3	GP1, GP17, GP66, GP68, GP71, EF212882
Hap_4	GP2, GP22, GP29, GP61, GP72, FM177761
Hap_5	GP3, GP8, GP13, GP24, GP30, GP36, GP37, GP38, GP51, GP54, GP64
Hap_6	GP4, GP6, GP70
Hap_7	GP5, GP10, GP12
Hap_8	GP7
Hap_9	GP14, GP25, GP28, GP57
Hap_10	GP15
Hap_11	GP16
Hap_12	GP18, GP19
Hap_13	GP23, GP27
Hap_14	GP31, GP39, GP60
Hap_15	GP33
Hap_16	GP35
Hap_17	GP52, GP65, GP67
Hap_18	GP53
Hap_19	GP58
Hap_20	AM711896

Table S4. Optimal data partitions and substitution models selected by partitionfinder for the BEAST analyses.

Partition	Ursidae Model	Skyline Model Selected	Skyline Model Used *	Composition
p1	TrN + G	TrN	TrN	12s, 16s
p2	K80 + G	HKY	HKY	ND1_CP1, cytb_CP1
p3	HKY + I	K80+I	K80	ND1_CP2, cytb_CP2
p4	TrN + I	HKY	HKY	ND1_CP3, cytb_CP3
p5	-	HKY+I+G	HKY+G	D-loop

* For some partitions, individual parameters of the partitionfinder selected models failed to converge satisfactorily, and so the number of free parameters were reduced in order to achieve convergence.

Table S5. Optimal data partitions selected by partitionfinder for maximum likelihood phylogenetic analysis.

Partition	Model	Composition
p1	GTR + G	12s, 16s, ND1_CP1
p2	GTR + G	ND1_CP2, cytb_CP2
p3	GTR + G	ND1_CP3, cytb_CP1
p4	GTR + G	cytb_CP3

Table S6. Variable sites in the aligned 655 bp D-loop data set.

	4	8	8	1	1	1	1	2	2	2	2	3	3	4	4	5	6	6
	2	7	8	1	3	3	4	0	2	7	7	7	7	0	7	3	1	2
				0	6	7	3	5	6	4	8	0	1	2	2	0	7	8
**GH01**	C	C	C	A	-	T	C	C	A	T	G	T	A	C	T	-	G	G
**GH02**	T	C	C	A	T	C	-	C	A	T	A	T	A	C	T	T	A	A
**GH03**	T	C	C	G	C	T	C	C	A	T	A	T	A	T	T	T	A	A
**GH04**	T	C	C	A	T	C	-	T	A	T	A	T	A	C	T	-	A	A
**GH05**	T	C	C	A	T	C	-	C	A	T	A	T	A	C	T	-	A	G
**GH06**	C	C	C	A	T	C	-	C	A	T	A	T	A	C	T	-	A	G
**GH07**	C	C	C	A	C	T	C	C	A	T	A	T	A	C	T	-	A	G
**GH08**	C	C	C	A	-	T	C	C	A	T	A	T	A	C	T	-	G	G
**GH09**	C	C	C	A	-	T	-	C	A	T	G	T	A	C	T	-	A	G
**GH10**	C	C	C	A	T	C	-	C	A	T	A	T	A	C	T	-	A	G
**GH11**	T	T	C	A	C	T	C	T	A	T	G	T	A	C	T	-	A	A
**GH12**	C	C	C	A	C	C	-	C	A	T	A	T	A	C	T	-	A	G
**GH13**	C	C	C	A	C	T	C	C	A	T	A	T	A	C	T	-	G	G
**GH14**	C	C	C	A	C	T	-	C	A	T	A	T	A	C	T	-	A	G
**GH15**	C	C	C	A	-	T	C	C	A	T	A	T	A	C	T	-	A	G
**GH16**	C	T	C	A	T	C	-	C	A	T	A	T	A	C	T	-	A	G
**GH17**	T	C	T	A	T	C	-	C	A	T	A	T	G	C	T	-	A	A
**GH18**	C	C	C	A	T	C	-	C	A	T	A	T	A	C	T	-	A	G
**GH19**	T	C	C	G	-	T	C	C	A	T	A	T	A	T	T	T	A	A
**GH20**	C	C	C	A	-	-	C	C	A	T	A	T	A	C	T	-	G	G
**GH21**	T	T	C	A	C	T	C	C	A	T	G	T	A	T	T	-	A	A
**GH22**	C	C	C	A	C	T	-	C	A	T	A	T	A	C	T	-	A	G
**GH23**	T	T	C	A	T	T	C	C	A	T	G	T	A	T	T	-	A	A
**GH24**	T	C	C	A	T	T	C	C	A	T	G	T	A	T	T	-	A	G
**GH25**	T	C	C	A	C	T	C	C	A	T	A	T	A	C	T	-	A	A
**GH26**	T	C	C	A	T	C	-	C	A	T	A	T	A	C	T	-	A	A
**GH27**	C	C	C	A	T	C	-	C	A	T	G	T	A	C	T	-	A	G
**GH28**	T	T	C	A	C	T	C	C	A	T	G	T	A	C	T	-	A	A
**GH29**	T	T	C	A	-	T	C	C	A	T	A	T	A	C	T	-	A	A
**GH30**	T	C	C	A	C	T	C	C	A	T	A	T	A	C	T	T	A	A
**GH31**	T	C	C	A	T	C	-	C	A	T	A	T	A	C	T	T	A	A

**GH32**	C	C	C	A	-	-	C	C	A	T	A	T	A	C	T	-	A	G
**GH33**	C	C	C	A	T	C	-	C	A	T	A	T	A	C	T	-	A	G
**GH34**	C	C	C	A	T	C	-	C	A	T	A	T	A	C	T	-	A	G
**GH35**	C	C	C	A	T	C	-	C	A	T	A	T	A	C	T	-	A	A
**GH36**	T	C	C	A	T	C	-	C	A	T	A	T	A	C	T	-	A	A
**GH37**	C	C	C	A	T	C	-	C	A	T	A	T	A	C	T	-	A	G
**GH38**	C	C	C	A	T	C	-	C	A	C	A	T	A	C	T	-	A	G
**GH39**	C	C	C	A	T	C	-	C	A	C	A	T	A	C	A	-	A	G
**GH40**	T	C	T	A	C	T	C	C	A	T	A	T	G	C	T	-	A	A
**GH41**	T	C	C	A	T	C	-	C	A	T	A	T	A	C	T	-	A	A
**GH42**	T	C	C	A	C	T	C	C	G	T	G	C	A	C	T	T	A	A

* Numbers in the first line indicate the nucleotide positon in 655 bp sequences.
